# Rapid eco-friendly synthesis, characterization, and cytotoxic study of trimetallic stable nanomedicine: A potential material for biomedical applications

**DOI:** 10.1016/j.bbrep.2020.100812

**Published:** 2020-10-05

**Authors:** Vivek K. Chaturvedi, Sachchida Nand Rai, Nazish Tabassum, Navneet Yadav, Veer Singh, Raghvendra A. Bohara, Mohan P. Singh

**Affiliations:** a Centre of Biotechnology, University of Allahabad, Prayagraj, 211002, India; b Department of Physics, University of Allahabad, Prayagraj, 211002, India; c School of Bio-Chemical Engineering, IIT-BHU, Varanasi, 221005, India; d CÚRAM, SFI Research Centre for Medical Devices, National University of Ireland, Galway, Ireland; e Centre for Interdisciplinary Research, D.Y. Patil Education Society (Institution Deemed to be University), Kolhapur, 416006, MS, India

**Keywords:** Trimetallic nanoparticles, Au–Pt–Ag, *Pleurotus florida* (Pf), Breast cancer, Nanomedicine

## Abstract

In the current scenario of the fight against cancer Integration of potential elements seems to be the best alternative since it overcomes the weaknesses of individuals and the combination of elements makes them formidable in the fight against the cancer war. Inspired by this objective and trusting our knowledge of paddy straw grown oyster mushroom, *Pleurotus florida* (Pf) mediated synthesis; a first-of-kind approach has been developed for the rapid synthesis of Au–Pt–Ag trimetallic nanoparticles (TMNPs). The developed method was successful, which was confirmed by Ultraviolet–Visible, X-ray diffraction, Transmission Electron Microscopy, Energy Dispersive Spectroscopy. Specifically, prepared TMNPs have been studied for their stability and size as a primary prerequisite for nanomedicine. Finally, the stable nanomedicine developed has been assessed for its performance against the highly metastatic breast cancer cell line (mda-mb-231). The performance was assessed using MTT assay and morphological readings, which were integrated with the cell viability data. We also determined the IC50 value, which was far superior to individual components and motivated us to postulate the possible breast cancer cell killing mechanism of TMNPs. The present study unlocks the new paths for the mushroom-mediated environmentally friendly, economic synthesis of trimetallic nanoparticles, which can be effectively used in cancer nanomedicine.

## Highlights

• Pleurotus florida (Pf) mediated synthesis was established for the first time.
• For rapid synthesis of Au–Pt–Ag trimetallic nanoparticles (TMNPs).
• TMNPs were characterized for physical, chemical properties and biological properties.
• The cell viability study shows TMNPs are potent anti-cancer agent (breast cancer cell line).

## 1 Introduction

In the last few decades, nanoparticles (NPs) have received much attention due to its unique properties for various applications such as health, energy conversion, waste minimization and degradation of pollutants in the industrial arena [1]. In human health, NPs based treatment has been in development for nearly two decades and has tremendously advanced in the delivery of therapeutic drugs, including peptides, proteins, and genes [2]. Today, with an increasing global population and needs, nanomedicine technology aims to increase the accuracy, effectiveness, protection, diagnosis and diagnosis and treatment of disease enforcement by exploiting the unique properties of engineered NPs [3].

At present, there are several famous elements for the preparation of metallic nanomaterials, such as gold (Au), silver (Ag), copper (Cu) and platinum (Pt) among them, Au nanomaterials, as a standard metal material, are especially favoured in biochemical and biomedical research due to their merits of stable product yield, versatile ligand alteration, and biocompatibility and the agreed role in drug delivery mechanism [4]. However, pure Au nanomaterials still exhibit shortcomings, such as high cost, relatively low thermal conductivity and optical instability which is directly correlated with the anticancer activity of the material. Pt element shows unique and powerful catalytic efficiency, but far from translational research into cancer therapy [5]. Besides, because of the limited Pt resource and rising cost as well as the difficulty of Pt nanomaterials in functional conjugation, there is still an urgent need to find substitutes for pure Pt catalyst.

In the past few years, synthesis and application of bimetallic and trimetallic NPs have drawn more attention because of their importance in optical, catalytic, and magnetic applications in various fields. Based on the above context, we envisage that it would be a good idea to combine Ag, Au, and Pt into a single nanoparticle, which is supposed to make up the disadvantages of the individual elements while realizing the fusion of their functions and advantages [6]. Gathering the merits of Au, Pt, and Ag will make it possible to integrate therapy effects and molecular recognition, thus laying a foundation for developing efficient and straightforward multifunctional theranostic nanoagents [7]. Multimetallic NPs show higher catalytic activities which makes them better in terms of biomedical applications as compared with monometallic NPs. In addition to this, an effort was made to reduce the expense of the TMNPs further, and a novel method was planned for this in the present manuscript by reducing the amount of Pt and Au for the synthesis TMNPs. Current work on multimetallic nanomaterials is in the initiation process, and our successful efforts have been to create a novel method for the production of trimetallic nanoparticles (TMNPs). More importantly, the work on the synthesis and biomedical application of Au–Pt–Ag TMNPs still has not been previously published, and our manuscript is the first kind of approach to the development of an eco-friendly synthesis and the study of its anticancer activity.

The eco-friendly synthesis of TMNPs Herein, it was proposed by oyster mushroom bio-based, eco-friendly synthesis [8]. The rapid development of nanoparticles is the reason behind using this method apart from eco-friendly synthesis [9]. TMNPs were fabricated with aqueous extract of *Pleurotus florida* (Pf) botanically species belonging to the genus *Pleurotus* called as ‘Dhingri’. Pf belongs from Pleurotaceae family contain nutraceuticals properties with many advantages like high protein content, low cost in production [10]. Capping and fabrication were possible by the binding of various bioactive compounds present in Pf *i.e.* phenols, proteins, amino acids, sugars, ketones, aldehydes, amines, and carboxylic acids [10]. The Pf-based biogenic approach is a better alternative to traditional physical and chemical methods. Hence, TMNPs, which have been considered a superior nanomaterial, hold great promise in cancer diagnosis and treatment [11]. However, to the best of our knowledge, there are no reports on combining green synthesis TMNPs with mushroom extract. At present, to concern with the biomedical applications such as cytotoxic studies, the present study is focused on the cytotoxic activity the *Pleurotus florida* mediated synthesis Trimetallic At-based nanocomposites for the first time on the MDA-MB-231.

## 2 Material and methods

All chemicals used in this investigation were reagent grade. All aqueous solutions were prepared in doubly distilled water. Hydrogen tetrachloroaurate (III) trihydrate (HAuCl_4_·3H_2_O), hexachloro platinic (IV) acid hydrate (H_2_PtCl_6_·xH_2_O) and silver nitrate (AgNO_3_) were purchased from Hi-media Laboratories Pvt. Ltd. Mumbai.

### 2.1 Culture and spawning of *Pleurotus florida*

The culture of Pf maintained on malt extract agar (MEA) medium at 25 °C ± 2 °C. Vegetative mycelium of Pf is referred to as spawn which was grown on wheat grains. Spawn was prepared by the following method of Sonam et al. [12]. Overnight soaked paddy straw was used as the substrate for the cultivation of mushroom, after that, for an hour paddy straw was again completely dipped in boiled water (temperature 70–80 °C) followed by draining out of excess water. Next step in spawning, 50 g of the wet weight of spawn was mixed with 1 kg of the substrate and filled into polythene bags. These methodologies is well established in the author's laboratory and has been detailed in Singh et al. [13].

### 2.2 Biosynthesis of Au–Pt–Ag NPs

**Fig. 1:**
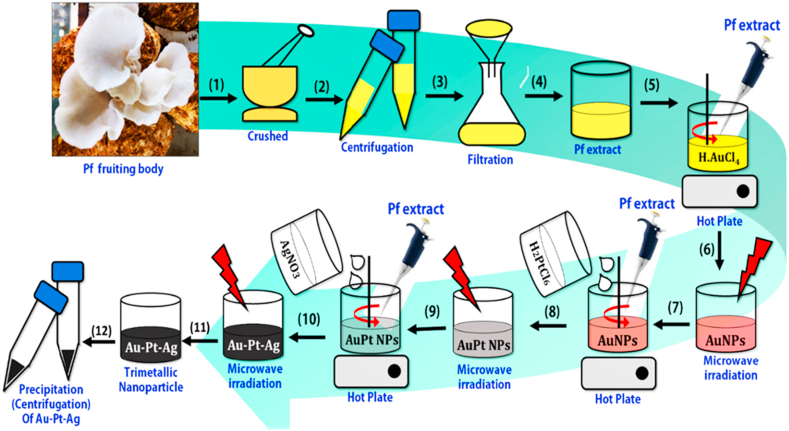
Schematic representation of the Au–Pt–Ag TMNPs synthesis route involving the extraction of the required phyto-components from the oyster mushroom (*Pleurotus florida*) and the microwave irradiation assisted synthesis of the Au–Pt–Ag nanoparticles.

Synthesis of TMNPs was performed using a one-step method that was developed in-house for rapid and uniform synthesis. For this microwave reduction method was used (Fig. 1). Briefly for one batch of synthesis of TMNPs 25% aqueous homogenate of the new fruiting body of *Pleurotus florida* (Pf) was prepared by stirred continuously for the overnight at 25±2 °C. Homogenate, centrifuged (500 rpm, 10min, four °C; C-24BL, Remi) and collected supernatant was passed through Whatman grade1 filter paper. 15 mL aqueous extract of PC was added drop by drop in 50 mL yellow aqueous solution of 0.01 M, HAuCl_4_·3H_2_O and reaction mixture was stirred followed by heating on the magnetic hot plate (60–80 °C) and microwaved (LG) (20s ON, 10s OFF). The synthesis of Au NPs was confirmed by the change in the colour of the solution was turned to slight yellow to brownish-purple. 50 mL of 0.01 M, H_2_PtCl_6_⋅6H_2_O and 50 mL of 0.01 M, AgNO_3_ was added respectively with 10 mL aqueous extract of PC into the colloidal solution of Au nanoparticles followed the constant stirring. This reaction mixture was microwaved for two minutes. The time of microwave irritation time was optimized previously (data not shown). The change in the colour of the solution into dark black confirms the synthesis of TMNPs. Then, the reaction mixtures were placed over magnetic starrier for 24 h, centrifuged at 12,000 rpm for 20 min (Eppendorf 5424) and pellets ready for further analysis [14,15].

### 2.3 Characterization of nanofluids

The developed TMNPs nanoparticles were subjected for preliminary optical properties by ultraviolet and visible absorption spectroscopy (analytikjena SPECORD®210 PLUS double beam spectrophotometer, nanoparticles were scanning over 200–800 nm). The particle size analysis of trimetallic nanofluids was determined by Zetasizer (Malvern UK) at room temperature in water and physiological pH. The particle size distribution was examined at a 90° scattering angle. For the morphology and particle size distribution, characterizations of nanoparticles were carried by Transmission electron microscopy (TEM) (Hitachi Tabletop Microscope Model TM 3000) equipped with SwiftED 3000 SDD detector for energy-dispersive spectrometry and elemental mapping, the size distribution of the nanoparticles measured by Image J software. The crystal structures were analyzed from SAED patterns recorded from TEM images. The purity of elemental compositions of the Au–Pt–Ag nanoparticles was examined by energy-dispersive spectrometry coupled with TEM. The crystalline nature of the nanoparticles was characterized by x-ray diffractometer (XRD, Proto A-XRD equipped with Cu Kα radiation λ = 1.5406 Å) in the range of 20°–80° with 2°/min scanning rate.

### 2.4 Cell culture

For the determination of cytotoxic analysis of TMNPs, the human breast cancer cell line (MDA-MB-231) cells were cultured in DMEM medium (10% FBS, penicillin, streptomycin) and maintained at a 37 °C with 5% CO_2_.

### 2.5 Cell viability assay

Cell viability assay of developed TMNPs was tested in vitro by using MTT and IC50 was determined for 24 h. Briefly, the 1 × 10^4^ MDA-MB-231 cells were seeded for testing were cultured on 48 well culture plate in DMEM containing ten wt% fetal bovine serum (FBS) and one wt% penicillin-streptomycin aqueous solution. After that, biosynthesized Au–Pt–Ag (20–100 μg/mL) was added in culture plates and incubated for 24 h. Cells were washed with PBS followed by the addition of 20 μL MTT dye (0.5 mg/mL) in each well of and incubated at 37 °C for 4 h. After removing all the culture medium, 150 μL DMSO was added per well to dissolve formazan crystals. The percentage of cell viability was measured on ELISA reader (Biotek Co., USA) at a wavelength of 570 nm (reference wavelength 630 nm) [16].

### 2.6 Statistical analysis

The data are presented as means ± standard deviation (SD).

## 3 Results and discussion

### 3.1 The developed method of TMNPs synthesis shows complete reduction and phase formation

**Fig. 2:**
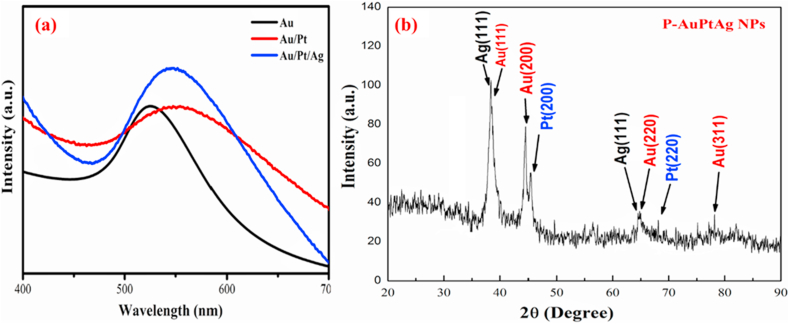
(a) Represent UV–Vis spectra of pure Au NPs, AuPt NPs, Au–Pt–Ag NPs at 524 nm, 554 nm and 547 nm respectively and synthesis of TMNPs from Pf mushroom extract; (b) Graphical illustration of the XRD patterns of Au–Pt–Ag nanocrystals. In this figure the peaks observed in the XRD patterns of TMNPs nanoparticles at 2θ values of 38.14°, 64.32°, 64.536°, 67.63° and 77.61°, match perfectly with the Ag (111), Ag (220), Pt (220) and Au (311) crystalline planes of the face-centred cubic structure of Au–Pt–Ag.

The developed in-house method has successfully ensured the TMNPs formation with uniform size and monodispersed. Our UV–Vis spectroscopy results confirm (Fig. 2a) the formation of TMNPs. It was noted that after the complete reduction of Au (gold) to the colloidal solution of Au–Pt–Ag, there was an immediate switch in the colour of the solution which was visually observed from yellow to brownish-purple and finally converted into black due to the clumping of Ag and Pt on the Au nanoparticle. The absorption peaks of Au NPs, AuPt NPs and Au–Pt–Ag TMNPs was recorded at 524 nm, 554 nm and 547 nm respectively which confirms the synthesis of TMNPs and the developed method was successful in the synthesis of TMNPs. This change in the colour transition from Au NPs to Au–Pt–Ag nanoparticles is attributed to shifting of the surface plasmon resonance (SPR) effects which are around 524 nm, 554 nm and 547 nm respectively. This shift in the band and the resulting colour changes confirms the complete reduction of the reaction mixture and the duration of the microwave irritation. In our optimization studies, we found that two minutes of irradiation, the reaction mixture is completely reduced and the TMNPs are synthesized. The shifting pattern from UV further supported our stepwise synthesis method. We observed the change in the shift in the other studies of Yadav et al. [17] reported the red, blue sifting of SPR band from 527 nm. Recently, Dlugaszewska et al. [15] reported the absorption peaks to come from 580 to 450 nm due to presence of Au and Ag in biosynthesized Au–Pt–Ag nanoparticle. Further compared our abortion band data with EDX analysis, reveals that the potential blue shift occurs because of the ratio of Ag in the reaction mixture is maximum in comparison to Au NPs, this observation supports the synthesis of Au-Pt-Ag [18].

XRD patterns of the TMNPs is shown in Fig. 2b, our XRD results further confirmed that the developed method was able to synthesis the pure phase of TMNPs. The synthesized material structure corresponds with the face centred cubic structure (FCC) confirmed from JCPDS of Au- 04–0784, Pt- 04–0802 and Ag- 04–0783. The figure indicates that a developed method does not impact the crystal structure of TMNPs. However, a slight decrease in the intensity was observed in the peaks, which are due to the presence of organic material on the surface, which induces microstrain. The crystallite sizes of TMNPs were calculated from FWHM of the most intense peak using the Debye–Scherrer formula. The average crystallite size was calculated 12 nm, which matches with the TEM of the sample [[18], [19], [20]].

### 3.2 TMNPS shows seven nm size with uniform distribution and nanocrystalline nature

**Fig. 3:**
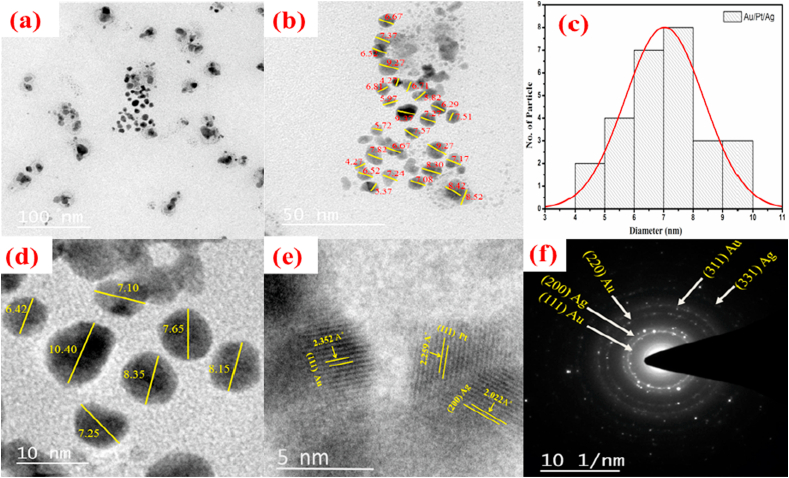
Transmission electron microscopy (TEM) micrograph of biosynthesized trimetallic Au–Pt–Ag nanofluids (TMNPs) by using *Pleurotus florida* (Pf) extract. (a) Trimetallic nanoparticles at 100 nm scale bar; (b) typical Representative image at 50 nm scale bar is a focused image of 100 nm scale; (c) Histogram plot for the particle size distribution and fitting Gaussian distribution curve is shown on the graph of TMNPs; (d) Size at 10 nm scale bar is a focused image of 50 nm scale; (d) SEAD patters of trimetallic nanofluids.

The developed particle was subjected to morphological analysis and size. Our TEM result suggests that (Fig. 3) Au–Pt–Ag trimetallic nanoparticles (TMNPs) appear uniform and spherical. The particle does not form agglomeration, and they are well dispersed (Fig. 3a). The obtained size range of trimetallic Au–Pt–Ag nanoparticles is 4–10 nm, and the mean diameter is 7 nm (Fig. 3b–d at the scale bar 50, 10 and 5 nm). More importantly, the obtained size is matching with the crystallite size. The SAED pattern (Fig. 3f) reveals Five bright circular rings with concentric spots from inner to outer, which orientation of crystal diffraction planes can be indexed as Au (1 1 1), Ag (2 0 0), Au (2 2 0), Au (3 1 1) and Ag (331) related to d-spacings of 2.34, 2.046, 1.441, 1.228 and 0.936 Å respectively confirming the face-cantered cubic (fcc) structure [21]. SAED pattern clearly shows the polycrystalline nature of biosynthesized Au–Pt–Ag TMNPs [17,22,23].

### 3.3 Elemental mapping shows Au in core while platinum and silver were uniformly distributed

**Fig. 4:**
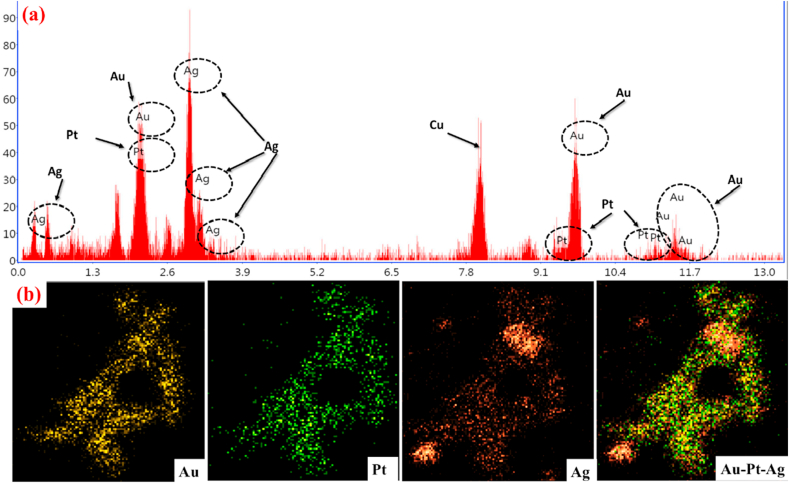
Confirms the (a) EDX spectra of TMNPs. (b) Elemental mapping of gold, silver, platinum and TMNPs are localized in specific areas within the precipitate. (For interpretation of the references to colour in this figure legend, the reader is referred to the Web version of this article.)

Our energy-dispersive X-ray (EDX) analysis of the TMNPs, shows a strong and clear metallic elemental signal of Au, Pt and Ag, which confirms the biosynthesis of TMNPs Fig. 4a. Besides, we also determined the concentrations of an individual element in our TMNPs. Similarly, our EDX analysis confirms UV results related to shifting of peaks during synthesis. TEM associated mixed and individual elemental mapping photographs of biosynthesized TMNPs shows in Fig. 4b. Elemental maps for Au, Pt and Ag metals were detectable, Au could be found in the core region, while platinum and silver were uniformly distributed. Our elemental mapping of all the nanoparticle showed that they were dispersed and some aggregation might be presented between metals. This presence finds were a similar result in corresponding to previously many research groups have reported elemental mapping of noble metals [17,24,25].

### 3.4 TMNPs shows stable nanofluid formation and monodisperse in nature

**Fig. 5:**
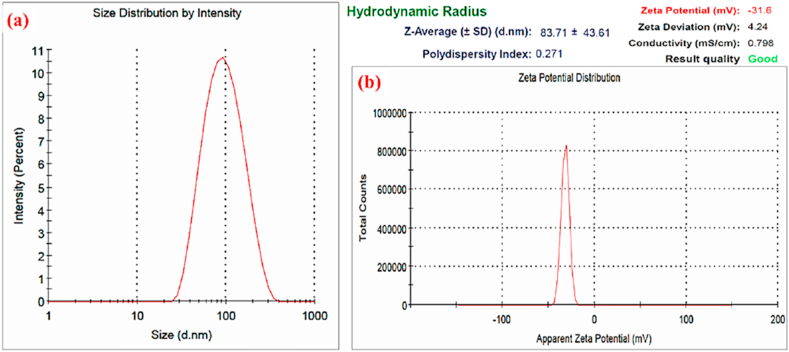
(a) Graphical representation of particle size distribution, hydrodynamic radius and polydispersity index of Au–Pt–Ag nanoparticles measured by Zeta sizer. (b) The zeta potential distribution graph showing negative zeta potential value and stable biosynthesized Au–Pt–Ag TMNPs.

The prerequisite for biomedical application is the formation of stable nanofluid formation. The prepared TMNPs were subjected to particle size analysis and stability study. Our particle size distribution plots of TMNPs determined by the dynamic light scattering (DLS) method depicted in Fig. 5. As is evident from the TEM and DLS studies on particle uniformity and monodispersity. These results are further supported by PDI measurements, which is 0.271 and suggests that the prepared TMNPs are monodisperse in nature. More importantly, the prepared particle was stable, and the mean surface charge or zeta potential was −31mV (Fig. 5 b). We also tested the stability for a week, and the prepared nanoparticles were stable. This may be one of the reasons why the particle shows better performance when it is introduced with cancer cells [[26], [27], [28]].

### 3.5 TMNPs a hope against triple-negative breast cancer

**Fig. 6:**
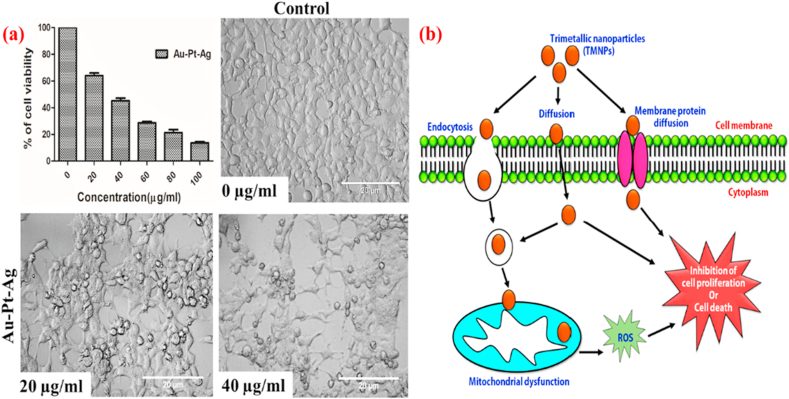
(a) Cytotoxic activity of TMNPs on MDA-MB-231 cell line (Percentage of cytotoxicity are presented as mean ± (SD). Morphological changes in MDA-MB-231 cells after the treatment of biosynthesized Au–Pt–Ag detected by phase-contrast microscope (Scale bar = 20 μm). **(b)** Proposed anticancer activity of trimetallic nanoparticles.

The possible the use of TMNPs is development as a possible nanomedicine to combat cancer. Our results of MTT assay confirm the toxicity of TMNPs against the Triple-negative Breast Cancer Cell Line (TNBC) MDA-MB-231 in a dose-dependent manner (Fig. 6a). Breast cancer is the second leading cause of cancer after lung cancer. Besides, almost 20% of breast cancer patients have TNBC cancer [29]. TNBC cells are derived from tumours that show the absence of ER, PgR, and HER2 receptor. More importantly, TNBC does not benefit from hormonal or trastuzumab-based therapies [30]. Hence, due to fewer treatment options available and lack of targeted therapies, this can be correlated to high moralities with TNBC in breast cancer family. Apart from surgery, platinum-based chemotherapy is the first choice, but due to limited success in platinum-based therapy, we focused on developing TMNPs, which are made up of Pt and further fortified with Au and Ag in the fight against TNBC [31].

Our study, which is presented in Fig. 6, clearly shows the success of our developed TMNPs in the killing the MDA-MB-231 cell line. Concentration-dependent cell death has been observed; the highest 10% cell viability has been recorded at 100 μg/mL concentration of nanoparticles. Besides, the IC_50_ value of Au–Pt–Ag was recorded at 40 μg/mL, which is motivating. From the morphology study observed that the treated cells depicted irregular shape and size comparison to control [[11], [32], [33]].

Au–Pt–Ag revealed potent anticancer against human breast cancer cell (MDA-MB-231). Previously many of research groups reported platinum having more catalytic activity in comparison of Au and Ag nanoparticles [34]. Here we correlate with the cell viability study and backing up with similar search we think that the TMNPs is causing cell death by increased ROS production which may be one of the possible mechanisms for such an elegant anticancer activity [14,34] (Fig. 6b).

## 4 Conclusion

In summary, best of our knowledge, for the first time we are reporting oyster mushroom *Pleurotus florida* mediated trimetallic nanoparticles synthesis via a rapid, simple, environmentally friendly, economically and feasible method. Our UV–visible and XRD data confirmed that the developed method is capable of synthesizing TMNPs, which is indeed a time-consuming process. More importantly, the developed method is ‘rapid’ and yields the particle size around eight nm. In physiological condition (pH) the system is super stable with monodispersity that further appeals for biomedical applications. Our study related to anticancer activity reveals that prepared nanomedicine is capable of killing triple-negative breast cancer cells with a superior IC 50 values. The present study unlocks the new paths for the mushroom-mediated environmentally friendly synthesis of TMNPs, which can be effectively used in cancer nanomedicine.

## Declaration of interests

The authors declare that they have no known competing financial interests or personal relationships that could have appeared to influence the work reported in this paper.

## Author contribution

Vivek K Chaturvedi: Formal analysis, Performed experiments, and analysis. Nazish Tabassum: Formal analysis, Performed experiments, and analysis. Navneet Yadav: Formal analysis, Performed experiments, and analysis. Veer Singh: Formal analysis, Performed experiments, and analysis. Sachchida Nand Rai Formal analysis, Data curation, helped in the analysis of the data. RB: Analysis of the results. Designing, correcting and writing of the manuscript.

## Declaration of competing interest

The authors declare no conflict of interest. GOIPD/2017/1283.
